# Acute effect of the COVID-19 pandemic on emergency transportation due to acute alcoholic intoxication: a retrospective observational study

**DOI:** 10.1186/s12199-021-01020-5

**Published:** 2021-09-30

**Authors:** Marina Minami, Kazumoto Kidokoro, Masamitsu Eitoku, Atsufumi Kawauchi, Masato Miyauchi, Narufumi Suganuma, Kingo Nishiyama

**Affiliations:** 1 Department of Environmental Medicine, Kochi Medical School, Kochi University, Nankoku, Kochi 783-8505 Japan; 2 Medical Course, Kochi Medical School, Kochi University, Nankoku, Kochi 783-8505 Japan; 3 Department of Health Policy, Kochi Prefectural Government, Kochi, Kochi 780-8570, Japan; 4 Department of Disaster and Emergency Medicine, Kochi Medical School, Kochi University, Nankoku, Kochi 783-8505 Japan

**Keywords:** Emergency transportation, COVID-19, Acute alcoholic intoxication

## Abstract

**Background:**

The COVID-19 pandemic has caused changes in people’s drinking habits and the emergency management system for various diseases. However, no studies have investigated the pandemic’s impact on emergency transportation for acute alcoholic intoxication. This study examines the effect of the pandemic on emergency transportation due to acute alcoholic intoxication in Kochi Prefecture, Japan, a region with high alcohol consumption.

**Methods:**

A retrospective observational study was conducted using data of 180,747 patients from the Kochi-Iryo-Net database, Kochi Prefecture’s emergency medical and wide-area disaster information system. Chi-squared tests and multiple logistic regression analyses were performed. The association between emergency transportation and alcoholic intoxication was examined. The differences between the number of transportations during the voluntary isolation period in Japan (March and April 2020) and the same period for 2016–2019 were measured.

**Results:**

In 2020, emergency transportations due to acute alcoholic intoxication declined by 0.2%, compared with previous years. Emergency transportation due to acute alcoholic intoxication decreased significantly between March and April 2020, compared with the same period in 2016–2019, even after adjusting for confounding factors (adjusted odds ratio 0.67; 95% confidence interval 0.47–0.96).

**Conclusions:**

This study showed that lifestyle changes due to the COVID-19 pandemic affected the number of emergency transportations; in particular, those due to acute alcoholic intoxication decreased significantly.

## Background

Acute alcoholic intoxication is a condition that is usually associated with the intake of large amount of alcohol [1]. At the medical institution to which the patient is transported, airway obstruction (positional asphyxia) due to their comatose state is often diagnosed, as well as severe cases of asphyxia due to vomiting [2].

The novel coronavirus disease (COVID-19) has spread globally and affected many countries [3] and regions [4], including areas in Japan [5]. The threat of the virus and governments’ measures—at both a national and prefectural level—to prevent the spread of the infection led to a substantial change in the normal movements of the population [6, 7]. Even before the state of emergency was declared, Japanese people refrained from organizing events or public gatherings and closed schools from early March 2020 [8, 9]. Most citizens also followed social distancing and some were even hesitant to go to hospitals or clinics due to the fear of getting infected [10]. The global health system faced adversity like never before. Changes in the emergency management system (EMS) as a result of society’s response to the COVID-19 pandemic have been observed in many countries [11, 12].

In the state of Missouri in the USA, the implementation of lockdown regulations significantly reduced the number of people with minor or no injuries due to traffic incidents, but not the number of people with serious injuries or deaths [11, 13]. The incidence of Takotsubo cardiomyopathy increased significantly in Ohio hospitals in March and April 2020, compared with previous years [12]. The percentage of emergency calls for youths with serious alcoholic intoxication in Trieste, Italy, was 2.96% in 2019, compared to 0.88% during the lockdown period and 11.3% after the lockdown [14].

Under pandemic, the biphasic nature of alcohol problems varies from region to region, with the possibility of increased consumption of alcohol due to stress or the possibility of decreased consumption of alcohol due to social restrictions on the other hand.

Globally, social changes during the pandemic-related lockdown also seem to have influenced people’s drinking habits [15]. However, there are very few studies currently available on the impact of COVID-19 on the EMS system; moreover, there are virtually no studies related to its impact on emergency transportation due to acute alcoholic intoxication.

Therefore, this study aims to examine the acute effect of the COVID-19 pandemic on emergency transportation due to acute alcoholic intoxication.

## Methods

### Study design and setting

In Japan, each municipality is responsible for the emergency services in its area; these services are activated when an individual in need of help dials 119. As of 2021, there are 15 fire departments in Kochi Prefecture. In the largest metropolitan area, Kochi City, there are 10 stations, in addition to the headquarters.

Kochi-Iryo-Net is Kochi Prefecture’s emergency medical and wide-area disaster information system (Kochi-Iryo-Net, 2021) [16]. Whenever the fire department provides emergency transportation service, the emergency team enters the information into the Kochi-Iryo-Net database. The information entered includes the name of the local fire department, the date and time of the call, the destination medical institution, and the distance from the fire department.

At the medical institution where the patient is transported to in an emergency, the degree of injury and the disease classification are entered by a doctor and all data are then aggregated by Kochi Prefecture.

**Fig. 1:**
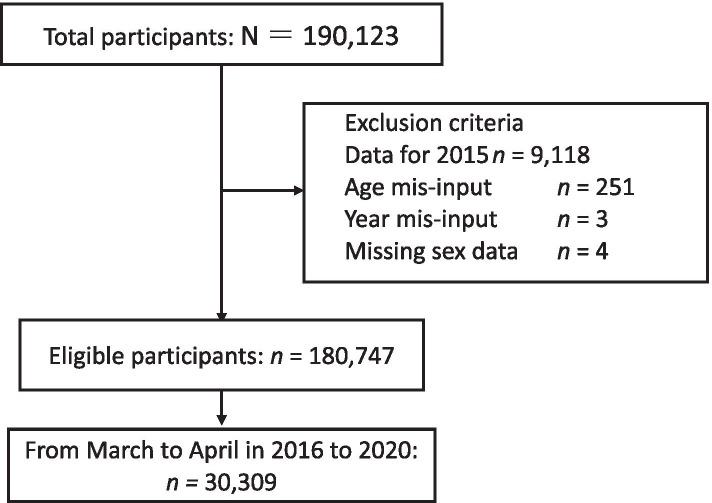
Flowchart of participant selection

Ambulance transportation data have been recorded and stored in Kochi-Iryo-Net from October 2015. When this study was conducted, the data from October 2015 to September 2020 was available. The database contains the transportation information of 190,123 patients. The data for transportations in 2015 (*n* = 9118) were excluded because the Kochi-Iryo-Net database started operating in October of 2015, and we could not make a comparison between 2015 and 2020 for the same months. Finally, this study included data of 180,747 patients for analysis. This study was a retrospective observational study that was performed using the opt-out method of Kochi-Iryo-Net’s website (Fig. 1).

### Data analysis

We performed a chi-squared test and a logistic regression analysis. To identify regions, we conducted the chi-squared test to determine the associations between the presence of acute alcoholic intoxication and the locations of fire stations, and patient sex and age. It should be noted that emergency transportation due to alcohol poisoning cannot be classified as such in the disease classification system because the system classifies it as an actual “poisoning.” We therefore chose “alcoholic intoxication” from among the names of injuries and illnesses listed, and the outcome was defined as “emergency transportation due to alcoholic intoxication.” A multiple logistic regression analysis was conducted for the outcome of emergency transportation due to alcoholic intoxication. This analysis measured the differences between the number of transportations during the voluntary isolation period (March and April 2020) and the same period in each year. We adjusted for the department (others, Kochi City as a reference), sex (female as reference), and age (older than 60 years as reference).

A two-tailed *p*-value of < 0.05 was considered to be statistically significant. All analyses were performed using Stata/MP 13.1 software (StataCorp., College Station, TX, USA).

## Results

**Fig. 2:**
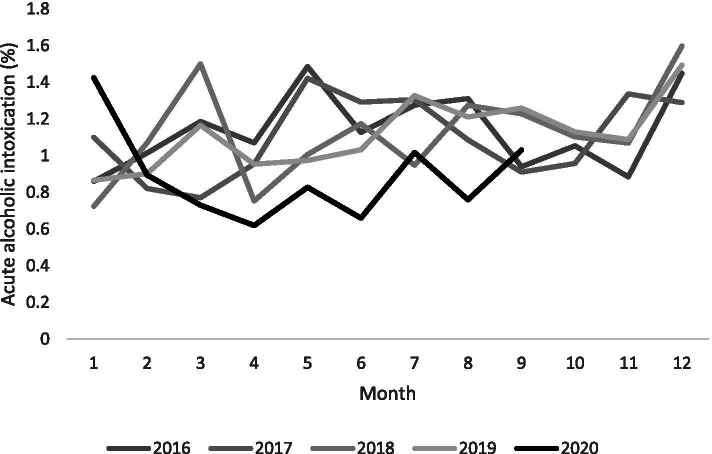
Monthly emergency transportation due to acute alcoholic intoxication, 2016 to September 2020. The *x* and *y* axes are emergency transportation due to acute alcoholic intoxication and monthly, respectively. The black line is transportation due to acute alcoholic intoxication in 2020

Figure 2 shows the monthly changes in the percentages of acute alcoholic intoxication among all emergency transportations in each year. The thick black line represents patients in 2020. The figure indicates that patients transported due to acute alcoholic intoxication in March and April of 2020 were fewer than those in other years.

Table 1 shows the baseline characteristics of emergency transportation in 2016–2019 and 2020. The number of emergency transportation due to acute alcoholic intoxication decreased in 2020. Approximately 1% of all emergency transportations was related to acute alcoholic intoxication. In 2020, emergency transportations due to acute alcoholic intoxication declined by 0.2%, compared with previous years. Also, compared to other years, younger age groups are experiencing a decrease in emergency transportation in 2020 (Table 1).

**Table 1:** Baseline characteristics and emergency transportation by 2020 and 2016–2019

Year	All	2016–2019	2020	
	180,747	154,049	26,698	
	*n* (%)			*p*-value
Department				0.65
Kochi City	78,635 (43.5)	67,054 (43.5)	11,581 (43.4)	
Others	102,112 (56.5)	86,995 (56.5)	15,117 (56.6)	
Sex				0.11
Male	89,068 (49.3)	75,791 (49.2)	13,277 (49.7)	
Female	91,679 (50.7)	78,258 (50.8)	13,421 (50.3)	
Age				<0.001
0–9	6044 (3.3)	5304 (3.4)	740 (2.8)	
10–19	5649 (3.1)	4934 (3.2)	715 (2.7)	
20–29	6950 (3.9)	6015 (3.9)	935 (3.5)	
30–39	7011 (3.9)	6066 (3.9)	945 (3.5)	
40–49	9866 (5.5)	8413 (5.5)	1453 (5.4)	
50–59	12,980 (7.2)	11,052 (7.2)	1928 (7.2)	
≥60	132,247 (73.2)	112,265 (72.9)	19,982 (74.8)	
Acute alcoholic intoxication	1968 (1.1)	1728 (1.1)	240 (0.9)	0.001

Table 2 shows baseline characteristic and emergency transportation due to acute alcoholic intoxication. Emergency transportation due to alcohol poisoning was more common in Kochi City (72.0%), the prefectural capital of Kochi Prefecture, and more common among men (67.4%) than women (32.6%), with those in their 20–29-year-olds (35.7%) being the most common age category.

**Table 2:** Baseline characteristic and emergency transportation due to acute alcoholic intoxication

Emergency transportation due to acute alcoholic intoxication	All	Acute alcoholic intoxication	No	
	*n* (%)			*p*
March–April				0.009
2020	5458 (3.0)	37 (1.9)	5421 (3.0)	
2016–2019	24,851 (13.8)	261 (13.3)	24,590 (13.8)	
Department				<0.001
Kochi City	78,635 (43.5)	1,417 (72.0)	77,218 (43.2)	
Others	102,112 (56.5)	551 (28.0)	101,561 (56.8)	
Sex				<0.001
Men	89,068 (49.3)	1327 (67.4)	87,741 (49.1)	
Women	91,679 (50.7)	641 (32.6)	91,038 (50.9)	
Age				<0.001
0–9	6044 (3.3)	0 (0.0)	6044 (3.4)	
10–19	5649 (3.1)	71 (3.6)	5578 (3.1)	
20–29	6950 (3.9)	702 (35.7)	6248 (3.5)	
30–39	7011 (3.9)	251 (12.8)	6760 (3.8)	
40–49	9866 (5.5)	246 (12.5)	9620 (5.4)	
50-59	12,980 (7.2)	254 (12.9)	12,726 (7.1)	
≥60	132,247 (73.2)	444 (22.6)	131,803 (73.7)	

Emergency transportation due to acute alcoholic intoxication decreased significantly in March and April 2020, compared with the same period in our reference year, 2016–2019 (*cOR* 0.64; 95% confidence interval [*CI*] 0.46–0.91). Even after adjusting for confounding factors, there was still a significant decrease (*aOR* 0.67; *95% CI* 0.47–0.96). Compared with other regions, Kochi City (*aOR* 2.59; *95% CI* 2.00–3.35) had significantly higher emergency alcohol transportations; the same applies to males, when compared with females (*aOR* 2.04; *95% CI* 1.59–2.61). In terms of age, there were significant differences in all age groups; compared with people older than 60 years, 20–29-year-olds (*aOR* 24.40; *95% CI* 18.01–33.06) had the highest rate of emergency transportation due to alcoholic intoxication (Table 3).

**Table 3:** Comparison of emergency transportations due to alcoholic intoxication

	Acute alcoholic intoxication			
	*cOR*	(*95% CI*)	*aOR*	(*95% CI*)
2016–2019	ref		ref	
2020	**0.64**	**(0.46–0.91)**	**0.67**	**(0.47–0.96)**
Department				
Kochi City			**2.59**	**(2.00–3.35)**
Others			ref	
Sex				
Men			**2.04**	**(1.59–2.61)**
Women			ref	
Age (years)				
0–9			Empty	
10–19			**2.13**	**(1.02–4.43)**
20–29			**24.40**	**(18.01–33.06)**
30–39			**8.49**	**(5.71–12.64)**
40–49			**5.28**	**(3.51–7.93)**
50–59			**4.17**	**(2.81–6.19)**
≥60			ref	

## Discussion

The results of our study showed that the number of emergency transportations due to acute alcoholic intoxication decreased in March and April of 2020, compared with the same period in other years (2016–2019).

In Japan, spring is the second most common season for alcohol intoxication emergencies, after the New Year holidays. No existing research has addressed the correlation between this excessive alcohol consumption and the need for emergency transportation due to alcoholic intoxication. Moreover, no graduation and entrance ceremonies or parties took place in 2020 and most of the usual spring events were scaled down or postponed due to COVID-19. We believe that these changes led to a decrease in alcohol-related emergencies. The decline in these numbers during the lockdown period is considered to be because of fewer outings. With fewer people going out and more people staying at home, the number of emergency transportations due to acute alcohol intoxication has decreased. March and April represent the end (or start) of the fiscal year in Japan, a time of joining, leaving, and transferring in schools and workplaces. During this time, parties are held, usually with alcohol, to welcome and send off people. At these parties, individuals are more likely to consume large amounts of alcohol and are often forced to drink, even non-drinkers and those who rarely drink consume alcoholic beverages. Early spring in Japan also brings cherry blossom viewing, which also contributes to the number of acute alcoholic intoxication cases.

The results show a clear decrease in the number of people who were transported by ambulance due to acute alcoholic intoxication in March and April of 2020, compared with other years. This may be because people were encouraged to stay home, to maintain social distancing, and not to visit restaurants that serve alcoholic beverages. During this extraordinary time, the transportation data gathered was unlike ever before. Since there were fewer transportations due to acute alcoholic intoxication in March and April of 2020—when people had less contact with each other—we believe that the risk of needing emergency transportation is larger at drinking parties in Kochi because people sometimes get overwhelmed in the moment and consume excessive amounts of alcohol, especially due to forced drinking and binge drinking [17, 18], rather than drinking by themselves.

The Kochi Prefecture Alcohol and Health Impairment Prevention addresses the environment and conditions surrounding alcohol use. The plan includes strategies for the prevention of alcohol-related health problems, early detection and treatment, and prevention of recurrence. In particular, to prevent alcohol abuse among juveniles and emergency transportation due to acute alcohol intoxication, it is necessary to provide education on alcohol-related health problems from childhood. Kochi Prefecture is promoting school education and awareness at home [19, 20]. To prevent the inducement of inappropriate drinking, it is also necessary to promote cooperation with alcoholic beverage vendors and to address the issue on a society-wide basis.

As soon as the period of self-isolation was relaxed, the suppressed movement of people seems to have bounced back and the number of alcohol-related incidents increased. The problem of acute alcoholic intoxication among young people is global and there are also reports of young people increasing the amount of alcohol they consumed during lockdowns [15]. This indicates the possibility that the decrease in emergency incidents due to alcohol intoxication is because of heavy drinking at home [21, 22]. Countermeasures need to be implemented to help people who are alcohol dependent due to unstable lifestyles or who are drinking more due to telecommuting.

The main strength of this study is its use of a large dataset from Kochi-Iryo-Net that contains more than 190,000 emergency transportation records from October 2015 to date. This system is designed to enter data when emergency transportation is dispatched, and it covers all emergency transportation data in Kochi Prefecture. The study also has several limitations. First, it is possible that there were patients with acute alcoholic intoxication who were not transported by ambulance; therefore, the data in this study do not represent all acute alcoholic intoxication cases in Kochi Prefecture. However, as fire stations throughout the target area were included, acute alcohol intoxication that requires urgent attention may have been identified. Second, transportation information was provided by ambulance crews, meaning that there was room for errors. There was a wide range of possible descriptions; for example, “acute alcoholic intoxication” and “neurological intoxication due to acute alcoholism.” However, the date, time, and other information were quite accurate because the paramedics used a bulletin board with their own system installed to enter this data. This unique system, which was mainly developed by Kochi Prefecture, uses tablets to collect information so that emergency transportation information can be captured and stored in real time. Third, this study used transportation records only in Kochi Prefecture; therefore, the results may not be generalizable to other prefectures. However, the fact that we were able to evaluate data from Kochi Prefecture, which has its own culture regarding alcohol consumption, is of some value.

Alcohol consumption by minors who are still growing up poses a number of physical and mental risks. To prevent underage drinking, it is necessary for the entire community, especially in Kochi Prefecture where drinking is tolerated, to ensure that minors understand the risks of drinking and to explain the risks to them at school and at home. In addition, during the New Year holidays, spring, and other times of the year when alcohol consumption increases, it is necessary to work together with businesses who serve alcoholic beverages to conduct campaigns to prevent excessive consumption and emergency transportation due to acute alcohol intoxication. In particular, young people need to be educated on how to avoid drinking in a way that can lead to acute alcohol intoxication, how to refuse alcohol if they are constitutionally incapable of drinking, and that they should never be forced to drink alcohol.

## Conclusion

This study showed that lifestyle changes due to the COVID-19 pandemic affected the number of emergency transportations—among these, the number of emergency transportations due to acute alcoholic intoxication—significantly. Considering our results, we suggest that people should refrain from forcing non-drinkers to consume alcohol and not allow the practice of “Ikki-nomi,” in order to reduce the number of emergency transports due to acute alcoholic intoxication at drinking parties, even after the pandemic is over and things get back to normal.

## Data Availability

The data that support the findings of this study are available from Kochi Prefecture, but restrictions apply to the availability of these data, which were used under license for the current study, and so are not publicly available. Data are however available from the authors upon reasonable request and with permission of the Kochi Prefecture government.

## Abbreviations

*aOR*: Adjusted odds ratio
EMS: Emergency management system
COVID-19: SARS-CoV-2 novel coronavirus disease
*CI*: Confidence interval
*OR*: Odds ratio
